# Pattern of adult intestinal obstruction at Tenwek hospital, in south-western Kenya

**DOI:** 10.11604/pamj.2015.20.31.5830

**Published:** 2015-01-13

**Authors:** Philip Blasto Ooko, Betty Sirera, Seno Saruni, Hillary Mariko Topazian, Russell White

**Affiliations:** 1 Department of Surgery, Tenwek Hospital, Bomet, Kenya

**Keywords:** Bowel obstruction, causes, outcomes, management, bowel gangrene

## Abstract

**Introduction:**

Acute mechanical intestinal obstruction (IO) is one of the leading causes of surgical admissions in most emergency departments worldwide. The causes of IO vary significantly depending on geographical location. The aim of this study was to identify the etiology, management and outcomes of patients with acute mechanical IO presenting in south-western Kenya.

**Methods:**

A 4 year (November 2009–October 2013) retrospective review of all adult patients admitted with acute mechanical IO at Tenwek Hospital in Bomet, Kenya.

**Results:**

A total of 303 male and 142 female patients, presented with acute mechanical IO during the study period. Mean patient age was 40.6 years (range 17-91), with peak incidence in those aged 31-40 years. The foremost signs and symptoms were abdominal pain (89.4%), abdominal tenderness (81.6%), vomiting (78%), abdominal distension (65.4%) and constipation (50.8%). Sigmoid volvulus (25.6%), adhesions (23.1%), small bowel volvulus (21.3%), and ileo-sigmoid knotting (8.5%) were the leading causes of IO. Laparotomy was undertaken in 361 (81.1%) cases, with bowel gangrene noted in 112 (30.4%). The overall morbidity and mortality rates were 15% and 4.5% respectively. Patients with gangrenous bowel at laparotomy had a higher morbidity rate (22.3% vs 9.6%, P=.001), a higher mortality rate (9.8% vs 3.2%, P=.02) and a longer duration of stay (9.9 days vs 7.6 days, P=.0001) compared to those with viable bowel.

**Conclusion:**

The most common causes of IO in this study were sigmoid volvulus, adhesions, small bowel volvulus and ileo-sigmoid knotting. Presence of bowel gangrene was associated with higher morbidity and mortality rates.

## Introduction

Intestinal obstruction occurs due to the failure of propagation of intestinal contents, and may be due to a mechanical or functional pathology. Acute mechanical IO is one of the leading causes of surgical admissions in most emergency departments’ worldwide [1], and is a significant cause of morbidity and mortality, especially when associated with bowel gangrene or perforation [2]. Data from Kenya on the epidemiology and outcome of IO is sparse. While the main causes of IO may vary from country to country or among regions within a specific country [3], knowledge of the local disease patterns and outcomes may raise the index of suspicion, and reduce delays in diagnosis, referral and/or operative intervention. This is especially helpful in settings with limited diagnostic modalities where making a definitive diagnosis may be challenging. The aim of this study was to evaluate the etiology, presentation, management strategies, and outcomes of patients with acute mechanical IO at a single institution over a four year period.

## Methods

A 4 year retrospective chart review of adult patients managed for IO at Tenwek Hospital, in Bomet, Kenya between November 1, 2009 and October 31, 2013. Tenwek is a 320 bed hospital that acts as a referral center for the south-western region of Kenya, holding a population of over 800,000 people. Cases were defined as patients aged = 17 years admitted with a diagnosis of acute mechanical IO. Charts with incomplete records or with functional causes of IO were excluded. Data concerning patient demographics, presenting signs and symptoms, X-ray findings, management, complications, outcomes, and duration of hospitalization were recorded from individual case records. The significance of any differences was assessed using a Fisher′s exact test or student's t-test where appropriate. P-values less than or equal to 0.05 were accepted as significant. The main outcomes of interest were the morbidity and mortality rates at discharge, and duration of hospitalization.

## Results

A total of 445 cases were gathered from the study period, comprised of 303 (68.1%) males and 142 (31.9%) females. The mean age was 40.6 years (range 17-91), and the peak incidence was noted in the 31-40 years age bracket (Table 1). The mean duration of symptoms was 3.1 days (range 1-21) with the majority (261, 58.7%) of patients reporting symptoms for 2 days or less. The most common signs and symptoms were abdominal pain (89.4%), abdominal tenderness (81.6%), vomiting (78%), abdominal distension (65.4%), constipation (50.8%), and peritonitis (30.3%). History of prior laparotomy was noted in 152 (34.1%) cases. A plain abdominal X-ray was ordered for most patients and reviewed by the on-call surgical team, but results were recorded in only 376 (84.5%) cases. The predominant findings recorded were small bowel distension with multiple air fluid levels (237, 63%), and large bowel distension (170, 45.2%). The major causes of IO, accounting for 78.5% of all cases, were sigmoid volvulus (25.6%), adhesive bowel disease (23.1%), small bowel volvulus (21.3%) and ileo-sigmoid knotting (8.5%) (Table 2). Obstructing large bowel tumors and incarcerated hernias constituted a minority of IO cases (2.5% and 1.1% respectively). Overall, the mortality rate at discharge was 4.5% and the average duration of hospitalization was 7.5 days (range 1-38). The majority of patients (60.2%) were discharged within 7 days, 34.8% within 8-14 days, and 4.9% after 14 days.

**Table 1:** Age distribution of patients (n = 445)

Age (years)	Cases (percentage)
17-20	52 (11.7%)
21-30	99 (22.2%)
31-40	107 (24%)
41-50	69 (15.5%)
51-60	48 (10.8%)
61-70	40 (9%)
>70	30 (6.7%)

**Table 2:** Distribution of IO causes according to total number of cases, management through laparotomy, and presence of bowel gangrene

Cause	Cases (n = 445)	Laparotomy (n = 361)	Bowel gangrene (n = 112)
Sigmoid volvulus	114 (25.6%)	103 (28.5%)	34 (30.4%)
Adhesions	103 (23.1%)	56 (15.5%)	5 (4.5%)
Small bowel volvulus	95 (21.3%)	95 (26.3%)	28 (25%)
Ileo-sigmoid Knotting	38 (8.5%)	38 (10.5%)	33 (29.5%)
Ascariasis	16 (3.6%)	6 (1.6%)	-
Intussusception	15 (3.4%)	15 (4.2%)	3 (2.7%)
Large bowel tumour	11(2.5%)	11 (3.1%)	-
Small bowel tumour	9 (2%)	9 (2.5%)	-
Incarcerated hernias	5 (1.1%)	5 (1.4%)	1 (0.9%)
Other	39 (8.8%)	23 (6.4%)	8 (7.1%)

### Management

Most patients (372, 83.6%) required laparotomy or endoscopic detorsion to relieve their obstruction. The main causes of IO in this group were sigmoid volvulus (SV) (114, 30.6%), small bowel volvulus (95, 25.5%), adhesive bowel disease (56, 15.1%) and ileo-sigmoid knotting (38, 10.2%). Fifty six patients with SV, without features suggestive of bowel gangrene, had successful rigid endoscopic detorsion. Subsequently, most of these patients (45, 80%), accepted to undergo definitive surgery for SV within the same hospitalization, while the remaining 11 declined surgery and were subsequently discharged.

A total of 361 patients underwent laparotomy. Bowel gangrene was noted in 112 (31%) cases. Sigmoid volvulus, small bowel volvulus and ileo-sigmoid knotting accounted for 84% of all cases of bowel gangrene (Table 2). The main operative procedures performed included resection and anastomosis (170, 47.1%), detortion and decompression (73, 20.2%), and adhesiolysis (51, 14.1%) (Table 3).

**Table 3:** Case distributions according to type of operative procedure performed (n = 361)

Procedure	Cases
Resection and primary anastomosis	170 (47.1%)
Detorsion and decompression	73 (20.2%)
Adhesionlysis	51 (14.1%)
Resection and colostomy	30 (8.3%)
Hernia repair	5 (1.4%)
Other	32 (8.9%)

At discharge, a total of 49 (13.6%) morbidities were noted in patients who underwent laparotomy, including surgical site infections (16, 4.4%), enterocutaneous fistula (4,1%), wound dehiscence (3, 0.8%), and intra-abdominal abscess (2, 0.5%). Nineteen deaths were noted during the admission period, leading to a postoperative mortality rate of 5.3%. Patients with gangrenous bowel at laparotomy had a higher morbidity rate (22.3% vs 9.6%, P=.001), a higher mortality rate (9.8% vs 3.2%, P=.02) and a longer duration of stay (9.9 days vs 7.6 days, P=.0001) compared to those with viable bowel.

Seventy three patients were successfully managed non-operatively, with the leading causes of IO being adhesive bowel disease (44, 60.3%) and worm obstruction (10, 13.6%). Non-operative management included maintenance of nil per os, intravenous fluids, and nasogastric tube drainage until bowel function returned. The mortality rate of patients undergoing non-operative management was 2.7% and the mean duration of hospitalization was 4.1 days (range 1-13).

## Discussion

IO remains one of the most common surgical diagnoses made in emergency departments worldwide [1], management of which requires quick, appropriate diagnosis and, rational and effective therapy [4]. In this series, the mean patient age (40.6 years) and a male predominance (68.1%) are consistent with other published studies from Africa, reporting mean ages between 32 and 45 years, and male predominance rates of 60-77% [3–7].

Significant regional variation in the etiology of IO has been noted amongst various African studies. Strangulated/incarcerated hernias and adhesive bowel disease accounted for 54-90% of all cases of IO in series from Nigeria [3, 5, 7, 8], Rwanda [4], and Sudan [6], while volvulus of the small or large bowel accounted for 55-74% of all cases of IO in series from Ethiopia [9, 10]. In agreement with the current study, one local study has revealed SV as the leading cause of bowel obstruction. Bahaty and Odhiambo [11], in a review of 90 cases of IO in adults and children in south-western Kenya, noted SV as the leading cause of bowel obstruction in 30% of cases, followed by obstructed/strangulated hernias in 18% and adhesions in 17%. Similarly, Tegegne [10], in a review of 139 patients managed operatively at a single institution in northern Ethiopia, noted the leading causes of IO to be SV (55.8%) and small bowel volvulus (SBV) (18.3%), with hernias and adhesions accounting for 11% of IO cases. Most other African studies report SV to occur at 6-15% of all cases of IO [3, 4, 6, 7].

The higher incidence of SBV as a cause of IO at Tenwek may be explained by the fact that this series only captured adults in whom SBV occurs more commonly than in children. Additionally, this subset of patients are more likely to be referred to our hospital due to the need for operative intervention, compared to other causes of IO that can be successfully managed non-operatively at lower level hospitals such as adhesive bowel disease. However, Kuremu and Jumbi [12], in a review of 263 cases of IO at a major referral hospital in western Kenya noted adhesive bowel disease to be the leading cause of IO, accounting for 40.7% of all cases. Incarcerated external hernias were rarely seen in this series (1.1%) compared to prior reported Kenyan series (18%) [11]. This may be due to the fact that obstructed hernias may have been managed at a patient's primary care facilities, and the predominant elective repair of symptomatic hernias at Tenwek before development of complications. Neoplasms, either of the small or large intestine are an uncommon cause of IO in many African series, reported at (2.2-9.14%) [3, 4, 9, 11]. Similarly, small and large bowel tumors caused 2% and 2.5% of all cases of IO respectively in this study.

The overall mortality rate in this series at 4.5% compares favorably with other published African series 6.7-20% [3–5, 7, 9]. Poor outcomes have been noted in patients who develop bowel gangrene [3, 9, 13], indicating the need for a high index of suspicion and early intervention, and/or referral to larger treatment centers in areas where the main causes of IO have a higher preponderance for development of bowel gangrene. The rate of bowel gangrene has been reported to be 93-100% in ileo-sigmoid knotting (ISK) [14, 15], 28-40% in SBV [3, 9], 24-46% in SV [13, 16–18], and 14-19% in adhesive bowel disease [3, 12]. Similarly, the incidence of bowel gangrene in patients undergoing operative management this series was 86.8% in ISK, 29.4% in SBV, 29.1% in SV and 8.9% in adhesive bowel disease. Thus SV, SBV and ISK accounted for a disproportionately higher incidence of bowel gangrene at 84.8% compared to the proportion of cases of IO undergoing laparotomy at 65.3%. Appropriate management, in this series, led to favorable outcomes in most patients.

## Conclusion

The most common causes of acute mechanical IO in this study were sigmoid volvulus, adhesive bowel disease, small bowel volvulus and ileo-sigmoid knotting. Presence of bowel gangrene at laparotomy was associated with more unfavorable outcomes, however patients can be managed successfully with low rates of morbidity and mortality. These results will inform primary care physicians working in Africa; encouraging a high index of suspicion for common causes of IO, leading to faster and better management and more favorable outcomes in patients with bowel obstruction.
